# Short-term effects and long-term changes of FUEL—a digital sports nutrition intervention on REDs related symptoms in female athletes

**DOI:** 10.3389/fspor.2023.1254210

**Published:** 2023-12-18

**Authors:** Ida Lysdahl Fahrenholtz, Anna Katarina Melin, Ina Garthe, Paulina Wasserfurth, Andreas Ivarsson, Siri Marte Hollekim-Strand, Karsten Koehler, Danielle Logue, Sharon Madigan, Maria Gräfnings, Monica K. Torstveit

**Affiliations:** ^1^ Department of Sport Science and Physical Education, University of Agder, Kristiansand, Norway; ^2^ Department of Sport Science, Linnaeus University, Växjö/Kalmar, Sweden; ^3^ The Norwegian Olympic and Paralympic Committee and Confederation of Sport, Oslo, Norway; ^4^ Department Health and Sport Sciences, School of Medicine and Health, Technical University of Munich, Munich, Germany; ^5^ School of Health and Welfare, Halmstad University, Halmstad, Sweden; ^6^ Department of Neuromedicine and Movement Science, Norwegian University of Science and Technology, Trondheim, Norway; ^7^ Sport Ireland Institute, National Sports Campus, Dublin, Ireland; ^8^ Department of Medical Science, Dalarna University, Falun, Sweden

**Keywords:** sports injuries, menstrual disturbances, low energy availability, endurance exercise, women's health

## Abstract

Female endurance athletes are at high risk for developing Relative Energy Deficiency in Sport (REDs), resulting in symptoms such as menstrual dysfunction and gastrointestinal (GI) problems. The primary aim of this study was to investigate effects of the FUEL (Food and nUtrition for Endurance athletes—a Learning program) intervention consisting of weekly online lectures combined with individual athlete-centered nutrition counseling every other week for sixteen weeks on REDs related symptoms in female endurance athletes at risk of low energy availability [Low Energy Availability in Females Questionnaire (LEAF-Q) score ≥8]. Female endurance athletes from Norway (*n *= 60), Sweden (*n *= 84), Ireland (*n *= 17), and Germany (*n *= 47) were recruited. Fifty athletes with risk of REDs (LEAF-Q score ≥8) and with low risk of eating disorders [Eating Disorder Examination Questionnaire (EDE-Q) global score <2.5], with no use of hormonal contraceptives and no chronic diseases, were allocated to either the FUEL intervention (*n *= 32) (FUEL) or a sixteen-week control period (*n *= 18) (CON). All but one completed FUEL and *n *= 15 completed CON. While no evidence for difference in change in LEAF-Q total or subscale scores between groups was detected post-intervention (BF_incl_ < 1), the 6- and 12-months follow-up revealed strong evidence for improved LEAF-Q total (BF_incl_ = 123) and menstrual score (BF_incl_ = 840) and weak evidence for improved GI-score (BF_incl_ = 2.3) among FUEL athletes. In addition, differences in change between groups was found for EDE-Q global score post-intervention (BF_incl _= 1.9). The reduction in EDE-Q score remained at 6- and 12- months follow-up among FUEL athletes. Therefore, the FUEL intervention may improve REDs related symptoms in female endurance athletes.

**Clinical Trial Registration:**

www.clinicaltrials.gov (NCT04959565).

## 1. Introduction

Sustainable and low-cost management of symptoms related to the syndrome Relative Energy Deficiency in Sport (REDs) is of key interest in female endurance athletes (1–3) due to the high reported prevalence of symptoms, ranging from 31% to 80% (4–8). The frequent occurrence of negative health and performance related consequences, including menstrual dysfunction with associated low bone mineral density and overuse injuries (3) calls for action. Insufficient energy intake relative to exercise energy expenditure, often denoted low energy availability (LEA), is the underlying etiological factor for REDs (3). The recommended treatment is therefore to increase energy intake, reduce exercise energy expenditure or a combination of both (9, 10). However, the evidence of intervention efficacy for the managements of REDs symptoms is limited and primarily based on case studies (11–13) and interventions without a control group (14, 15), or in non-competitive females (16). Successful nutrition interventions have suggested that future studies implementing strategies to provide more personalized dietary interventions accounting for food preferences, dietary patterns across the day, timing of food intake and macronutrient composition may have the potential to be more effective (16).

The menstrual cycle is an energy demanding process, involving hormonal synthesis and follicular development, and eumenorrhea is recognized as an important health indicator for female athletes (17). Therefore, menstrual function is a frequently used marker when screening for LEA and REDs and assessing the safety of female athletes’ sports participation (4, 10, 18). In fact, it has been suggested that assessing self-reported symptoms of LEA, including menstrual function, provides a better assessment of the overall health status of an athlete compared to a snapshot of current energy availability, where assessment of dietary intake and exercise energy expenditure is susceptible to several sources of errors (6, 19, 20). The Low Energy Availability in Females Questionnaire (LEAF-Q) (4), where the main emphasis is laid on menstrual function, is one of the most frequently used screening tools for detecting female athletes at risk of LEA and REDs (20). The LEAF-Q is validated in endurance athletes (4) and this is also where one of the highest prevalence of menstrual dysfunction is reported in sports ranging from 0% to 20% for primary amenorrhea (late menarche), 0%–56% for secondary amenorrhea (no bleeding for minimum of three consecutive menstrual cycles), and 0%–39% for oligomenorrhea (<9 menstrual bleedings per year), depending on the diagnostic method used (21). Although the infertility associated with menstrual dysfunction in athletes may be transient (22), prolonged or severe LEA and the multiple metabolic and endocrine alteration associated with menstrual disturbances e.g., elevated cortisol and lowered estradiol, insulin and T3 levels can have serious negative impact on bone health via an estrogen-dependent and estrogen-independent pathway, which may be irreversible (23, 24). Low bone mineral density constitutes an increased risk for bone stress injuries, resulting in long absences from sport participation (25, 26), emphasizing the need for prevention at all levels (27).

Negative gastrointestinal tract function has also been associated with LEA and REDs (3). More specifically, persistent LEA can result in mucosal atrophy characterized by diminished intestinal function and morphological changes including decreased villous height, crypt depth, surface area, and epithelial cell numbers (28). In addition, LEA and REDs have been associated with an excessive dietary fiber intake among female endurance athletes (29). The gastrointestinal problems may appear as delayed gastric emptying, bloating, constipation, and increased intestinal transit time (3). Gastrointestinal problems, commonly reported by endurance athletes, may not only be detrimental to health and quality of life, but also to athletic performance (30, 31).

As formulated by Ackerman et al. (32) “*It is time for a drastic paradigm change in women's sport, coupled with education at all levels to improve the long-term health and athletic achievement of female athletes*” [(32), p.1 line 15–19]. One of the proposed steps in the management of REDs is to raise awareness of the negative effects of LEA so athletes can make wise decisions for their own long-term health (32). In essence, inadequate knowledge of optimal sports nutrition and the negative health and performance consequences of LEA, coupled with a normalization of REDs symptoms, e.g., menstrual dysfunction, appears to be frequent underlying causes of LEA (33, 34). Though, adequate nutrition knowledge is necessary for optimal nutrition habits, it may not be a sufficient factor for ensuring a true change in nutritional behavior in athletes (35). Furthermore, motivation, enabling, and supporting athletes have been identified as additional components necessary for changes in nutritional behavior (33). We have previously reported strong evidence for improved sports nutrition knowledge along with weaker evidence for increased energy intake in female endurance athletes with risk of REDs after a 16-week sports nutrition intervention, consisting of online sports nutrition lectures combined with individual athlete-centered nutrition counseling (the FUEL study) (36).

Parallel to measure physiological symptoms that may be affected by a nutrition intervention, it is important to monitor psychological symptoms associated with LEA. This includes eating disorder symptoms. Nutrition education and counseling have been reported to increase eating disorder symptoms in young ballet dancers (37, 38). Hence, although LEA and REDs occurs frequently without disordered eating behavior or eating disorders (39–41), one may fear that a nutrition intervention aiming at increasing energy intake may pose a risk for the development of eating disorders in an already high-risk group (42). Furthermore, there is a reported association between symptoms of eating disorders and exercise addiction (43) and REDs may therefore be associated with exercise addiction in endurance athletes (39, 44).

Therefore, the primary aim of the present analysis was to investigate immediate effects (pre-post intervention) and long-term changes (6- and 12-months follow-up) of the FUEL intervention study, on common symptoms associated with REDs; menstrual and gastrointestinal function and injuries. Secondary, the aim was to investigate any symptoms related to the risk of eating disorders and exercise addiction. Specifically, the goal of this analysis was to investigate whether the LEAF-Q, Eating Disorder Examination (EDE-Q), and Exercise Addiction Inventory (EAI) scores change differently from the pre- to postintervention in the intervention group compared to the control group, and to investigate how the symptoms develop up to 12-months follow-up in the intervention group.

## 2. Methods

The study design, recruitment process, and intervention content have been described in detail elsewhere (36). The study was approved by the regional ethics committee in Norway (31,640), Sweden (2019-04809), and by the Norwegian Centre for Research Data (968,634) and registered at www.clinicaltrials.gov (NCT04959565). Originally, the study was planned and approved to include a wide range of REDs related clinical biomarker measurements and a control group prior to initiation of the intervention. Due to the COVID-19 pandemic the first round of recruitment had to be cancelled. Further, since all physical contact with the participants was prohibited, the final design and measures are strongly influenced by the pandemic restrictions. Consequently, all medical procedures were excluded in the final research plan, thus, the study did not need an additional ethical approval at the other study sites (Germany and Ireland).

### 2.1. Study design

This was a multicenter study recruiting female endurance athletes from Norway, Sweden, Ireland, and Germany. Athletes were seasonally allocated to the FUEL intervention (FUEL) or a control condition (CON). The intervention group received weekly online lectures in sports nutrition combined with individual athlete-centered nutrition counseling with an experienced sports nutritionist for sixteen weeks. The control group received no lectures or counseling. The study was initiated with a screening phase, where athletes completed an online survey via the data collection tool Nettskjema that was connected to the safe Services for Sensitive Data (TSD) platform (University of Oslo). In this part of the study, athletes provided background information and completed the LEAF-Q (4), EDE-Q (45), and the Exercise Addiction Inventory (46). Athletes with risk of LEA, defined as a LEAF-Q score ≥8 (4), and low risk of disordered eating behavior, defined as an EDE-Q global score <2.5 (47), were invited to participate in the study. Athletes completed the same questionnaires after the 16-week FUEL/CON condition. In addition, the FUEL intervention group completed a 6- and 12-months follow-up answering the LEAF-Q, the EDE-Q, and the EAI. Other assessments included in the study, including diet and training log, has previously been described and analyzed (36).

### 2.2. Eligibility criteria

Eligibility criteria for the study were (1) competitive female endurance athlete, (2) 18–35 years of age, (3) training ≥5 times/week, (4) no use of hormonal contraceptives for at least six weeks prior to the study, (5) no chronic disease (e.g., Crohn's disease or hypothyroidism) or diagnosed menstrual dysfunctions not related to LEA (e.g., polycystic ovarian syndrome or endometriosis), (6) non-smoker, (7) not pregnant or planning a pregnancy, (7) speaking/understanding Norwegian, Swedish, English, or German.

### 2.3. Recruitment

Athletes were recruited from November 2020 to September 2021 via Norwegian, Swedish, Irish, and German competitive endurance sports clubs, coaches in endurance sports at the Olympic sports center in Norway and via social media with a link to the project website. The recruitment targeted summer endurance disciplines (runners, orienteers, cyclists, and triathletes) during November/December with the initiation to the intervention in January, while the recruitment targeted winter endurance disciplines (biathletes and cross-country skiers) in May with the initiation to the intervention in June.

In total, 208 participants signed up for the study. Of these, 141 were excluded: *n* = 2 male athletes; *n* = 2 < 18 years; *n* = 1 > 35 years.; *n* = 1 badminton player; *n* = 3 with chronic diseases (*n* = 1: Crohn's disease, *n* = 1: Hashimoto's thyroiditis, *n* = 1: hypothyroidism); *n* = 55 hormonal contraceptive users; *n* = 23 with a EDE-Q global score ≥2.5; *n* = 51 with a LEAF-Q score <8, and *n* = 3 for not providing any contact information. The LEAF-Q responses of *n* = 67 athletes were analyzed in more detail, and some were contacted to clarify their answers. This resulted in *n* = 7 athletes being excluded due to a suspected false positive identification of the risk of LEA. Further, *n* = 4 athletes were unavailable, *n* = 3 responded too late in relation to intervention start-up and allocation to sports nutritionists, and *n* = 3 athletes declared severe illness ahead of the baseline measurements (i.e., abdominal surgery and COVID-19). In total, *n* = 18 athletes, who had signed up during their competition season, were allocated to a 16-week waiting control condition (CON) of which *n* = 15 athletes completed (*n* = 1 wanted to start using hormonal contraceptives, while we were unable to contact *n* = 2). In total, *n* = 32 athletes were directly allocated to the FUEL intervention, while *n* = 1 terminated participation in the project in week 13 due to experiencing too much work related to the project. Consequently, *n* = 31 (97%) completed the FUEL intervention and *n* = 15 (83%) completed the CON condition.

#### 2.3.1. Final inclusion of participants in the analyses

One athlete in FUEL missed the postintervention survey relevant for this paper (but completed the other measurements), while all participants in CON completed the survey with the LEAF-Q, EDE-Q, and EAI pre- and postintervention. Consequently, *n *= 30 and *n *= 15 athletes were included in the analyses comparing pre- and post-measurements for FUEL and CON, respectively. Twenty-six of the 30 FUEL athletes completed the 6-months follow-up. In terms of the LEAF-Q analysis, *n *= 3 had started using hormonal contraceptives, *n *= 1 reported pregnancy/breastfeeding and was therefore excluded from the 6-months follow-up. Twenty-three FUEL athletes completed the 12-months follow-up. Additional *n *= 2 had started using hormonal contraceptives and *n *= 1 reported pregnancy/breastfeeding and was therefore excluded in the 12-months follow-up for the LEAF-Q analyses.

### 2.4. Nutrition intervention

The 16-week intervention consisted of weekly online lectures in sports nutrition targeting female endurance athletes with risk of REDs, combined with individual athlete-centered nutrition counseling every other week.

The sixteen sports nutrition lectures integrated evidence-based sports nutrition information and recommendations. They were developed by four researchers and practicing sports nutritionists, initially in Norway and Sweden, including a comprehensive manuscript for each session, and subsequently translated into English and German. All sixteen lectures were comprehensively reviewed and finally approved by all four researchers/sports nutritionists. The recorded lectures had a mean duration time of 25.0 ± 8.4 min. Key topics were information about REDs, the importance of the menstrual cycle for health and performance, macronutrient recommendations for endurance athletes, and nutritional periodization. Every week during the intervention, participants received an e-mail with a link and password to the lecture of the week located on a closed online platform. Participants had the opportunity to watch the lectures when suitable during their everyday lives and to watch them repeatedly if they wanted.

The nutrition counseling was administrated via the teleconferencing platform Zoom, Zoom Video Communication, Inc. (California, USA). The first consultation was scheduled to run for 1.5 h (actual duration: 73 ± 15 min), while the following seven consultations were scheduled to run for approximately 1 h (actual duration: 55 ± 6 min). The team of counsellors, consisted of three Norwegian, four Swedish, two Irish, and one German highly experienced sports nutritionists, who work with Elite athletes on a daily basis. Self-determination theory was chosen as a core foundation for the FUEL counseling, since this approach has been found to be effective in promoting behavior change (48). An athlete-centered, empathic communication approach, inspired by core skills in motivational interviewing (49) was utilized.

### 2.5. Measures and instruments

#### 2.5.1. Low energy availability in females questionnaire

The validated screening tool LEAF-Q (4) was used to assess self-reported symptoms of LEA; injury frequency the past year, current gastrointestinal function, and current and past reproductive function. The LEAF-Q is validated in female endurance athletes and has a total of 9–25 questions depending on the respondent's answer, including those related to hormonal contraceptive use. A total score ≥8 was considered at risk of LEA (4). Athletes completed the LEAF-Q at pre- and postintervention/control period, and also at 6- and 12-months follow-up for the FUEL group. Because a LEAF-Q score ≥8 was used as an inclusion criterion, all athletes in the present study had a LEAF-Q score ≥8 at pretest. Since the LEAF-Q assesses injuries the past year, the injury score was considered less important at postintervention measurement. Similarly, it is not possible to change the answer to some of the questions related to menstrual function during a 16-week period [“*How old were when you had your first period?*” and “*Did your first menstruation come naturally (by itself)?*”]. Therefore, it was of interest to look at possible changes on single questions related to menstrual function, namely: “*Do you have normal menstruation?*” and “*Do you experience that your menstruation changes when you increase your exercise intensity, frequency or duration?*” Minor clarifications from the original LEAF-Q were added and has been described previously (39).

#### 2.5.2. Eating disorder examination questionnaire

The EDE-Q was used to measure behavioral and cognitive symptoms of eating disorders the past 28 days (45). It has been validated in an athletic population (50) and is a frequently used screening tool for disordered eating and LEA/REDs (20). The EDE-Q consists of 28 items which can be divided into four subscales (restraint, eating concern, shape concern, and weight concern) and a global score averaging the subscales, used as cut-off for eating disorder pathology. In the present study, a global EDE-Q score ≥2.5 was used to classify athletes with disordered eating behavior (39, 47, 51). Because an EDE-Q global score <2.5 was used as an inclusion criterion, all included athletes had an EDE-Q global score <2.5 at pretest.

#### 2.5.3. Exercise addiction inventory

The EAI was used to assess symptoms of exercise addiction with a score ≥24 considering participants at risk of exercise addiction (46, 52). The EAI consists of six general components describing the degree of addiction rated on a five-point Likert scale: salience (exercise is the most important thing in life), conflicts (e.g., interpersonal conflicts due to the exercise behavior), mood modification (a coping strategy to regulate emotions), tolerance (increasing amounts of exercise is needed to achieve effect), withdrawal symptoms (e.g., irritability when an exercise session is missed), and relapse (reversions to earlier patterns). Originally, the EAI was validated in recreational exercisers but has later been validated in elite athletes (53).

### 2.6. Statistics

Data analyses were conducted using JASP (version 0.17.1.0). All analyses were conducted within the Bayesian statistical framework (54, 55). Descriptive statistics were expressed as frequencies with percentage for binary and categorical data and as means ± standard deviation (SD) for continuous data. Group comparisons for baseline characteristics were conducted using Bayesian Independent Samples *t*-test for normally distributed data and Mann–Whitney test for non-normally distributed data. Bayesian contingency table tests were used to compare groups for categorical data. Within-group differences from pre- to postintervention were investigated using Bayesian Paired Samples *t-*test for normally distributed data and with Wilcoxon Signed-Rank test for non-normally distributed data. Group comparisons from pre- to postintervention were conducted using a Bayesian repeated measures analysis of variance (ANOVA) with default priors and compared to the null model. Non-normally distributed data were transformed using SPSS [version 28.0.1.1 (14)] but did not change the interpretations of the results compared to analyzing the non-transformed data. A group × time interaction effect was hypothesized, i.e., that the FUEL and CON group's LEAF-Q scores would change differently over time (alternative hypothesis). To calculate the Bayes Factor (BF) for the interaction effect only inclusion probabilities for matched models were considered (55). BFs between 1 and 3 were considered to indicate weak evidence for the alternative hypothesis, BFs between 3 and 10 were considered moderate evidence for the alternative hypothesis, while BFs greater than 10 were considered as strong evidence for the alternative hypothesis (56). Menstrual function for individual questions in the LEAF-Q was analyzed in a descriptive manner due to insufficient number of participants. Within FUEL group comparisons for LEAF-Q, EDE-Q, and EAI scores for the four measurement time points (pre-, postintervention, 6- and 12-months follow-up), were conducted using a Bayesian repeated measures ANOVA. Menstrual function for individual questions in the LEAF-Q was analyzed in a descriptive manner.

## 3. Results

Endurance athletes from Norway (*n* = 11), Sweden (*n* = 17), Ireland (*n *= 5), and Germany (*n* = 12) were included from the following endurance disciplines: running (*n* = 14), orienteering (*n* = 7), triathlon (*n *= 12), cycling (*n *= 5), cross country skiing (*n* = 1), and biathlon (*n* = 6). Participant characteristics are presented in Table 1. There was no evidence of statistical differences when comparing the two groups’ baseline characteristics (BFs < 1).

**Table 1:** Participant characteristics divided by intervention (FUEL) and control (CON) groups.

	FUEL (*n *= 30)	CON (*n *= 15)
Age (years)	25.2 ± 4.09	24.1 ± 4.7
Height (cm)	169.5 ± 6.3	171.2 ± 7.1
Body weight (kg)	59.6 ± 7.1	59.3 ± 5.0
Body Mass Index (kg/m^2^)	20.7 ± 2.1	20.3 ± 1.7
Training volume (h/month)	45.2 ± 16.5	47.0 ± 18.9
Full-time athlete (%)	16.7	20.0
Level of competition		
Club (%)	63.3	86.7
National team (%)	20.0	6.7
Professional (%)	10.0	6.7
Others (%)^a^	6.7	0.0

FUEL, the FUEL intervention group; CON, the control group.

Continuous data are presented as mean ± SD and categorical data as percentage.

^a^ Athletes who did not identify themselves as competing at club-, national team-, or professional level, e.g., competing in one of the endurance disciplines but not affiliating within a club.

### 3.1. Symptoms of low energy availability

#### 3.1.1. Comparing pre- and postintervention group differences

The FUEL athletes reduced the LEAF-Q total score from 12.0 ± 2.8 to 9.8 ± 4.3 (BF_10_ = 20.92) compared to CON athletes reducing the LEAF-Q total score from 11.0 ± 3.0 to 10.3 ± 2.5 (BF_10_ = 0.79) with no evidence for difference in change between groups (Table 2). Nor did any of the changes in the LEAF-Q subscale scores differ between groups as indicated by the lack of an interaction effect (BF_incl_ < 1). At posttest, total LEAF-Q score was <8 for *n *= 11 (37%) of the FUEL athletes and *n *= 2 (13%) of the CON athletes (BF_10_ = 1.267).

**Table 2:** Low energy availability in females questionnaire scores pre- and postintervention.

	FUEL (*n* = 30)			CON (*n* = 15)			Difference in change between groups, BF_incl_
	Pre	Post	Difference pre-post	Pre	Post	Difference pre-post	
LEAF-Q total score	12.0 ± 2.8	9.8 ± 4.3	−2.2 ± 3.6	11.0 ± 3.0	10.3 ± 2.5	−0.7 ± 1.7	0.850
Injury score	3.2 ± 2.3	2.8 ± 2.3	−0.3 ± 2.0	3.7 ± 2.0	3.5 ± 2.1	−0.3 ± 1.4	0.320
Gastro-intestinal score	2.3 ± 2.1	1.7 ± 1.5	−0.6 ± 1.6	2.3 ± 1.7	2.1 ± 1.5	−0.1 ± 1.6	0.544
Menstrual score	6.6 ± 2.5	5.3 ± 3.0	−1.2 ± 1.8	5.1 ± 2.7	4.7 ± 2.3	−0.4 ± 2.2	0.729

Data are presented as mean ± SD.

BF_incl_, bayes factor for inclusion of group × time interaction; CON, control group; FUEL, the FUEL intervention group, LEAF-Q, low energy availability in females questionnaire.

**Figure 1:**
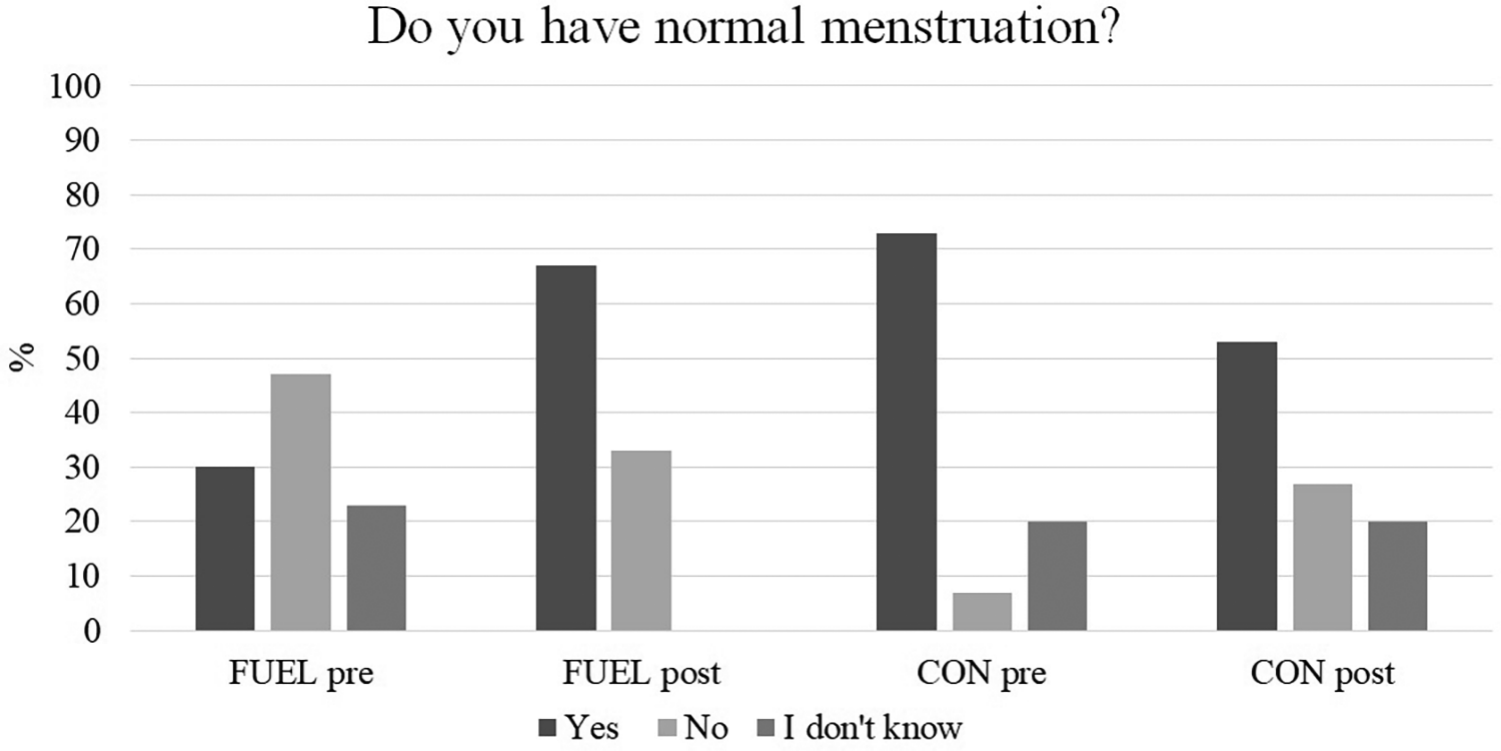
Self-reported eumenorrhea from the low energy availability questionnaire pre-and postintervention. Data are presented as percentages. CON, the control group; FUEL, the FUEL intervention group.

The number of participants that reported eumenorrhea increased among FUEL athletes from 30% (*n *= 9 athletes) at pretest to 67% (*n *= 20 athletes) at posttest and decreased among CON athletes from 73% (*n *= 11) to 53% (*n *= 8) (Figure 1). Five of the 14 (36%) FUEL athletes, who reported menstrual dysfunction at pretest, reported eumenorrhea at posttest. Of the FUEL athletes who reported menstrual dysfunction at pretest and eumenorrhea at posttest, all reported their latest menstruation within the last 0–3 month at pretest. Three FUEL athletes and one CON athlete reported secondary amenorrhea at pretest. None of them improved their menstrual function form pre to posttest.

**Figure 2:**
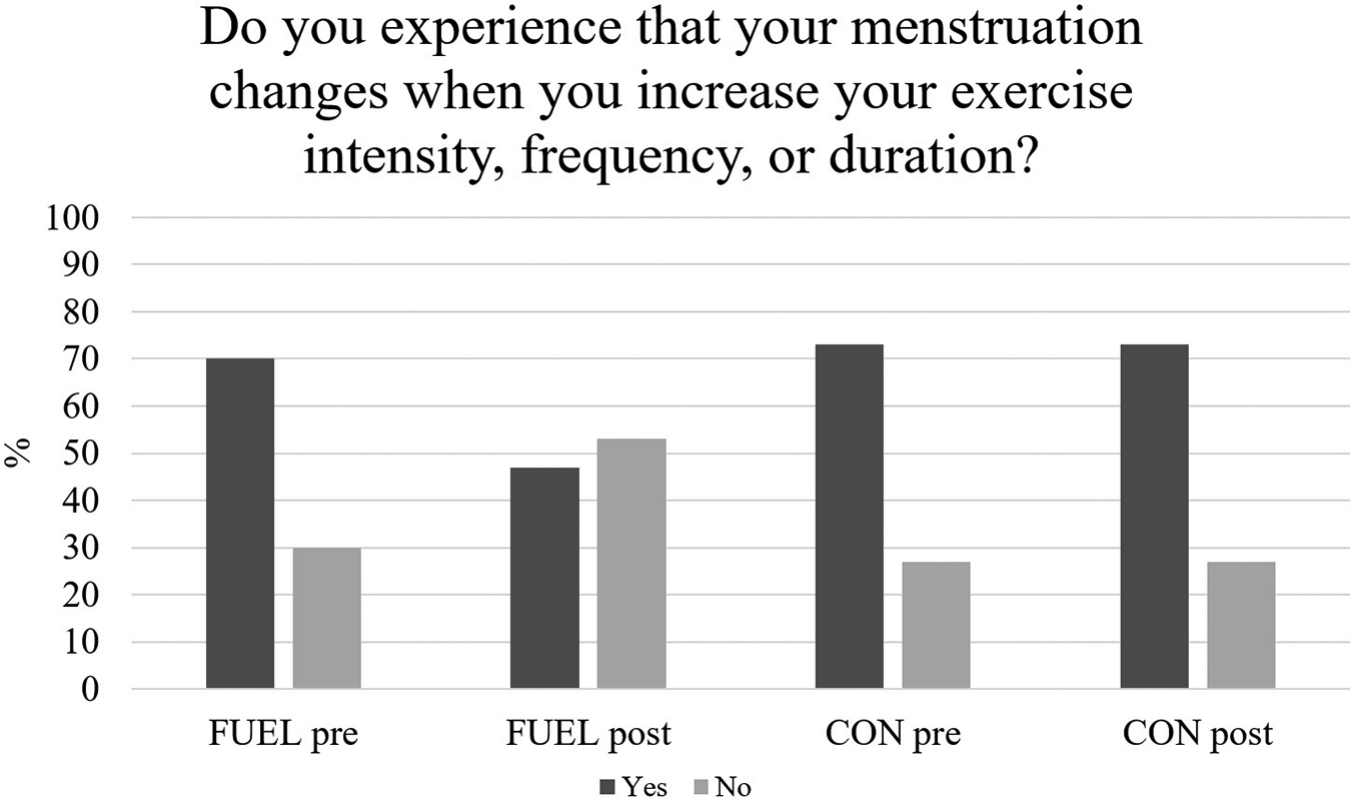
Reduced, or absence of, menstrual bleedings with increased training load from the low energy availability questionnaire pre-and postintervention. Data are presented as percentages. CON, the control group; FUEL, the FUEL intervention group.

Seven (23%) FUEL athletes and three (20%) CON athletes were unaware whether they had normal menstruation at pretest. All FUEL athletes were able to define whether they had normal menstruation or not at posttest, while the number was unchanged among CON athletes. The number of athletes who reported reduced or absence of menstrual bleedings with increased training load decreased from *n *= 21 (70%) to *n *= 14 (47%) among FUEL athletes while the number was unchanged among CON athletes (*n *= 14/73%) (Figure 2). Twelve (40%) FUEL athletes and four (27%) CON athletes reported late menarche a (menarche after 15 years of age).

#### 3.1.2. Six- and 12-months follow-up

**Figure 3:**
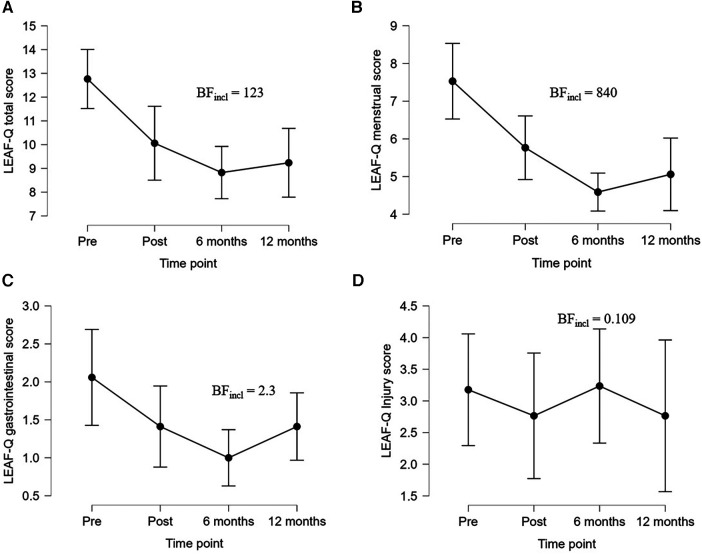
Changes in LEAF-Q (**A**) total score, (**B**) menstrual score, (**C**) gastrointestinal score, and (**D**) injury score for the FUEL athletes at pre- and postintervention, and at 6- and 12-months follow-up. Data are presented as mean and 95% credible intervals. BF_incl_, bayes factor for inclusion of time interaction, LEAF-Q, low energy availability in females questionnaire.

Six- and 12-months follow-up revealed strong evidence for improvement in LEAF-Q total score for FUEL athletes comparing the three (BF_incl_ = 441) and four (BF_incl_ = 123) measurement points, respectively (Figure 3A). This was explained by improvements in the menstrual score (6-months: BF_incl_ = 4,486, 12-months: BF_incl_ = 840) (Figure 3B) and the gastrointestinal score (6-months: BF_incl_ = 9.5, 12-months: BF_incl_ = 2.3) (Figure 3C). We found weak evidence for an improvement in the gastrointestinal score from 6- to 12-months follow-up (BF_10 _= 1.2) while no evidence for improvement in LEAF-Q total score, menstrual score or injury score when comparing 6- and 12-months follow-up (BF_10 _< 1) (Figure 3D).

At 6-months follow-up, 45% of FUEL athletes had a total LEAF-Q score <8, and 21% at 12-months follow-up. The two FUEL athletes with secondary amenorrhea at pretest, who was eligible for long-term follow-up, still had not improved menstrual function at 6 months follow-up, but one reported eumenorrhea at 12-months follow-up.

### 3.2. Symptoms of disordered eating behavior

#### 3.2.1. Comparing pre- and postintervention group differences

The EDE-Q global score decreased from 1.03 ± 0.73 to 0.72 ± 0.69 (BF_10_ = 11.84) among FUEL athletes and was unchanged among CON athletes (0.80 ± 0.74 at pretest and 0.96 ± 0.85 at posttest, BF_10_ = 0.41) with weak evidence for a difference in change between groups as indicated by the interaction effect of BF_incl = _1.858 (Table 3). The largest within-group difference among FUEL athletes for the EDE-Q subscales was detected for the restraint subscale score (BF_10_ = 14.87). In contrast, weak evidence for an increase in the EDE-Q subscale weight concern was found among controls (BF_10_ = 1.68).

**Table 3:** Eating disorder examination questionnaire scores pre- and postintervention among FUEL (*n *= 30) and CON athletes (*n *= 15).

	FUEL (*n* = 30)			CON (*n* = 15)			Difference in change between groups, BF_incl_
	Pre	Post	Difference pre-post	Pre	Post	Difference pre-post	
EDE-Q global	1.03 ± 0.73	0.72 ± 0.69	−0.31 ± 0.76	0.80 ± 0.74	0.96 ± 0.85	0.16 ± 0.56	1.858
EDE-Q restraint	0.79 ± 0.94	0.38 ± 0.62	−0.41 ± 0.98	0.96 ± 1.21	0.83 ± 1.08	−0.16 ± 0.51	0.498
EDE-Q eating concern	0.72 ± 0.75	0.46 ± 0.62	−0.25 ± 0.64	0.39 ± 0.39	0.51 ± 0.45	0.12 ± 0.30	2.006
EDE-Q weight concern	1.18 ± 0.85	0.97 ± 0.90	−0.21 ± 0.96	0.92 ± 1.08	1.4 ± 1.3	0.46 ± 1.08	1.768
EDE-Q shape concern	1.42 ± 0.94	1.05 ± 0.95	−0.37 ± 1.02	0.98 ± 0.65	1.16 ± 1.11	0.18 ± 0.83	1.230

Data are presented as mean ± SD.

BF_incl_, bayes factor for inclusion of group × time interaction; CON, the control group; FUEL, the FUEL intervention group; EDE-Q, eating disorder examination questionnaire.

The EDE-Q global score increased above the 2.5 threshold post-intervention for two (7%) FUEL athletes (pre-intervention EDE-Q global scores of 0.4 and 2.4, respectively) and one (7%) CON athlete (pre-intervention EDE-Q global score 2.4) to EDE-Q global scores 2.8, 3.0, and 2.5, respectively.

#### 3.2.2. Six- and 12-months follow-up

**Figure 4:**
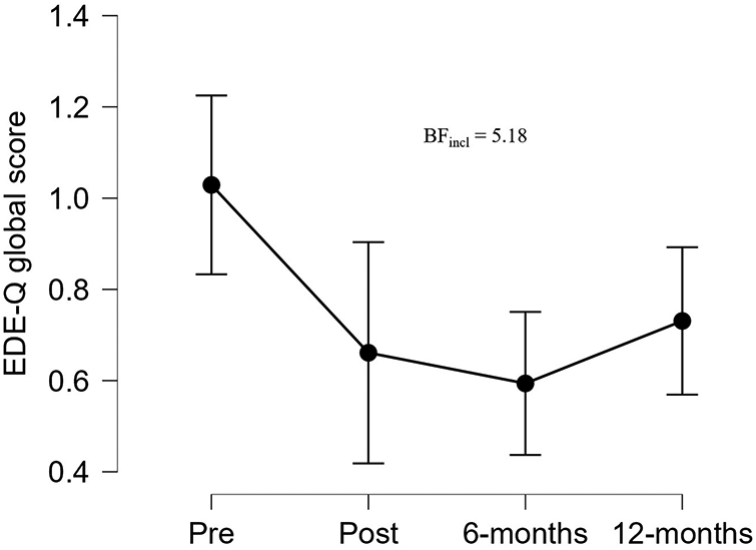
Eating disorder examination questionnaire global score for the FUEL athletes at pre- and postintervention, and at 6- and 12-months follow-up. Data are presented as mean and 95% credible intervals. BF_incl_, bayes factor for inclusion of time interaction; EDE-Q, eating disorder examination questionnaire.

Long-term follow-up revealed moderate evidence (BF_incl_ = 5.18) for reduced EDE-Q global score for FUEL athletes comparing all four measuring points (Figure 4). The largest reduction in the EDE-Q subscale scores was seen in the restraint subscale (BF_incl_ = 16.45).

The two FUEL athletes with EDE-Q global scores ≥2.5 at postintervention, had EDE-Q global scores of 0.0 and 0.3, respectively, at 6-months follow-up and 0.3 and 0.8, respectively, at 12-months follow-up.

### 3.3. Exercise addiction inventory

#### 3.3.1. Comparing pre- and postintervention group differences

Within group analyses revealed no evidence for changes from pre- to posttest in EAI total or the six item scores among FUEL nor CON athletes (BF_10_ < 1). Nor did we find evidence for difference in change between groups for the EAI total score (FUEL pre: 20.7 ± 3.0, FUEL post: 20.8 ± 2.7 vs. CON pre: 20.6 ± 3.0, CON post: 21.1 ± 2.9) or any of the six item scores (BF_incl _< 1).

#### 3.3.2. Six- and 12-months follow-up

**Figure 5:**
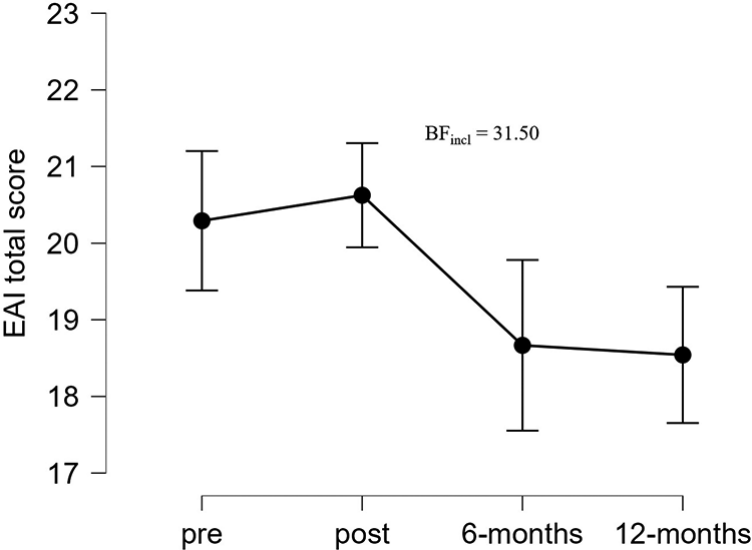
Exercise addiction inventory total score for the FUEL athletes at pre- and postintervention, and at 6- and 12-months follow-up. Data are presented as mean and 95% credible intervals. BF_incl_, bayes factor for inclusion of time interaction; EAI, exercise addiction inventory.

Six- and 12-months follow-up revealed strong evidence (BF_incl_ = 31.50) for reduced EAI total score for FUEL athletes comparing the four measuring points (Figure 5).

## 4. Discussion

To our knowledge, this is the first study to explore changes on several REDs related symptoms in female endurance athletes with risk of REDs after a nutrition intervention using validated screening tools, comparison with a control group, and inclusion of long-term follow-up. More specifically the current study explored changes in menstrual and gastrointestinal function, injuries, eating disorder and exercise addiction symptoms. The FUEL study was an international multicenter study with weekly online sports nutrition lectures combined with individual consultations every other week. The lectures were specifically designed for female endurance athletes with risk of REDs. Although no evidence for difference in change between FUEL and CON athletes pre- to postintervention were found in LEAF-Q scores, long-term follow-up revealed strong evidence for reduced LEAF-Q total and menstrual scores among FUEL athletes. Importantly, the nutrition intervention did not result in negative effects related to eating disorder or exercise addiction symptoms. Rather, there was evidence for improved EDE-Q scores after the FUEL intervention. The reduction in eating disorder symptoms for FUEL athletes remained at 6- and 12- month follow-up.

In this study, athletes were categorized with risk of REDs using the LEAF-Q (4). The LEAF-Q has been validated in female endurance athletes, 18–39 years of age, training ≥5 times/week with Cronbach's Alpha 0.61–0.79, and an acceptable sensitivity (78%) and specificity (90%) (4), making it a good alternative to assess symptoms of LEA in this group of athletes. The LEAF-Q has subsequently been validated in a mixed sport-cohort (*n *= 75, 18–32 years), which demonstrated high sensitivity for the detection of low bone mineral density and menstrual dysfunction, suggesting that injury and menstrual function cutoff score also may be appropriate in mixed-sport cohort (57). The researchers concluded that LEAF-Q total score <8 can be used to determine females at low risk of LEA related conditions given the high negative predictive values identified in this study (57). In the present study we examined all LEAF-Q responses in detail and excluded athletes who had been diagnosed with menstrual dysfunction not related to LEA and others where false positive identification of problematic LEA was expected (e.g., athletes who had been involved in a bicycle crash and menstrual dysfunction in the past resulting in a LEAF-Q total score ≥8). Nevertheless, using screening tools as inclusion and exclusion criteria contains a risk of including false positive cases (e.g., high LEAF-Q total score due to acute injuries), including false negative cases (e.g., athletes reporting eumenorrhea while undetected subclinical menstrual dysfunction (23, 58), as well as excluding false negative cases (e.g., high menstrual function score due to polycystic ovarian syndrome while coexisting symptoms of LEA).

In our study, the decline in LEAF-Q total score was 18% among FUEL and 6% among CON athletes. Although there was a lower risk rate of LEA among FUEL (64%) compared to CON (87%) athletes at posttest, we did not detect between group difference from pre- to post intervention when comparing LEAF-Q total score. The 16-week intervention period may have been too short to detect differences in the measured symptoms. Especially regarding the LEAF-Q injury score, which is related to the previous year and associated with low bone mineral density (4, 57), where an improvement cannot be expected within the time frame of the study period (59). But the absence of intervention effect may also be attributed to the time required to restore normal menstrual function and the complexity of changing eating habits.

All FUEL athletes who reported menstrual dysfunction at pretest but eumenorrhea at posttest, had reported a recent bleeding at pretest, while none of the three FUEL athletes reporting long-term absence of bleeding at pretest, had improved menstrual function at posttest. The two FUEL athletes with long-term absence of bleeding, who were eligible for long-term follow-up, still had not improved menstrual function at 6-months follow-up, but one reported eumenorrhea at 12-months follow-up, suggesting that recovery time from more severe menstrual disorders may be longer. Previous studies have reported mean time to restoration of menstruation to be as high as 16 ± 3 months among college athletes with nonpharmacologic therapies (60) and researchers have suggested that the time required to resume menstruation depends to a large extend on the starting point, including the duration of the menstrual dysfunction (14, 15). Unfortunately, the maximum duration of menstrual dysfunction assessed in the present study was ≥6 months (corresponding to the response option when answering “*no*” to “*do you have normal menstruation*”). In the present study six athletes, all with menstrual dysfunction, reported bone stress injuries during the last year at pretest, indicating long-term exposure of LEA.

Indeed, habits may take more than sixteen weeks to change (61) and we have previously emphasized the complexity of improving eating habits in this group of athletes (36). Since the increase in energy intake was modest among FUEL athletes (138 ± 453 kcal/day, corresponding to an increase of only 5%) (36), it may have been insufficient for improving REDs related symptoms in some of the athletes. The five FUEL athletes who reported menstrual dysfunction at pretest and eumenorrhea at posttest had a slightly higher increase in energy intake compared to the nine FUEL athletes who reported menstrual dysfunction both at pre- and posttest (7% vs. 1%). Previous studies with athletes and active females have reported increase in energy intake of 17% (14) and 18% (15, 16) after nutrition interventions of 6, 9, and 12 months, respectively. In these studies, 88% (14) and 23% (15) of the athletes restored regular menstruation after the intervention, while De Souza et al. reported improved menstrual function in 64% in a group of active females (16). Although we recognized the complexity of habitual changes in the study planning phase and implemented individual athlete-centered nutrition counseling, sixteen weeks may be too short for changing eating habits that can result in improvement of REDs symptoms. Since energy availability is energy intake relative to exercise energy expenditure, changes in LEAF-Q score could also be attributed to changes in training volume. We have previously reported decreased training volume among FUEL and CON athletes from pre to posttest with no difference in change between groups (36). Hence, training load was reduced independent of group and athletic season. Although training adjustment was not a part of the FUEL intervention, it is possible that some athletes deliberately have reduced their training volume to improve REDs symptoms. While the relative increase in energy intake may be crucial, it has been suggested that increase in body fat mass is an important predictor of restoration of menstrual function in athletes and active females (15, 16). Unfortunately, body composition was not possible to measure in the present study due to the COVID-19 pandemic (39).

At pretest ∼20% of the participants in the present study were unable to define their menstrual status while all FUEL athletes could define their menstrual status at posttest, with unchanged results for CON athletes, suggesting that the FUEL intervention succeeded in increasing the awareness of the menstrual cycle. Being aware of one's menstrual cycle, and the importance of having a regular menstrual cycle, seems like an obvious first step in the prevention of problematic LEA and REDs for female athletes. Especially since menstrual dysfunction is associated with low bone mineral density reported in 17%–45% of female endurance athletes (6, 40, 62–64) and an increased risk for bone stress injuries, resulting in long absences from sport participation (25, 26).

Among FUEL athletes the 6- and 12-months follow-up revealed strong evidence for improvement of LEAF-Q total score explained by improvements in the gastrointestinal score and in particular the menstrual score. Although positive changes in LEAF-Q total and subscale scores were observed, menstrual-, injury-, and total scores were all above the suggested cut-offs [≥2 for injuries, ≥2 for gastrointestinal symptoms, ≥4 for menstrual function, and ≥8 for total score (4)] at all four measuring points. At 6-months follow-up, 45% had a LEAF-Q score <8, while only 21% at 12-months follow-up. These findings may indicate that these female athletes need continuous nutritional support (e.g., individual follow-up sessions).

Importantly, no adverse effects on eating disorder or exercise addiction symptoms were found after participating in the FUEL intervention. Rather, we found evidence for a difference in change between groups for the EDE-Q global score, while long-term follow-up for FUEL athletes suggested persistent reduction in EDE-Q global and a reduction in EAI score. A recent systematic review of eating psychopathology interventions delivered to athletes (65, 66) found that less than half of the included studies reported sustained reductions in eating psychopathology, while two studies on ballet dancers reported an increase in eating psychopathology symptoms following the interventions. Importantly, our study differentiates from the studies in the systematic review by excluding athletes with risk of eating disorders, since these athletes are recommended an interdisciplinary treatment including psychiatric treatment (66). Interestingly, the authors of the review conclude that future interventions should investigate other modes of delivery beyond face-to-face group sessions, including digital approaches, which makes intervention retention more flexible for the participants but also serve to overcome stigma (65). This may in part explain the positive development in EDE-Q scores among FUEL athletes in the present study, but the explanation may also be found in the principles of the FUEL intervention reflected in the teaching videos and the individual consultations: Focus away from body weight and more towards food as fuel and that there are no “good” or “bad” foods. Two FUEL athletes had increased EDE-Q global score above the 2.5 cut-off from pre- to posttest which reduced well-below the 2.5 at long-term follow-up indicating that some athletes may have transient changes in eating disorder symptoms during the athletic seasons, and that regular screening and follow up assessments are needed.

Among FUEL athletes, post-hoc tests found weak to moderate evidence for change comparing the preintervention EAI total score with 6- and 12-months follow-up, respectively. Although no difference in changes in EAI scores between FUEL and CON athletes were detected postintervention, it is interesting that FUEL athletes reduced their score at long-term follow-up. This should be seen in light of the reduced LEA and eating disorder symptoms at 6- and 12-months follow-up, symptoms that have been reported to be associated with symptoms of exercise addiction (39, 43, 44). However, both changes in athletic season (67) and the COVID-19 pandemic (68) may also be explanatory factors to the changes in exercise addiction symptoms. Further, FUEL athletes with risk of primary exercise addiction preintervention, reduced and increased LEAF-Q total score, respectively, suggesting a complex symptom picture and the potential interaction between exercise addiction and risk of REDs.

### 4.1. Strengths and limitations

A strength of the present study is the combined intervention design, including both online lectures and individual consultations, which were athlete-centered and aimed at inducing long-term behavioral change by enabling female athletes to actively formulate nutritional and behavioral goals to support their own long-term health, as researchers have requested (32). As previously recommended (16), this type of intervention opens for a more individual-centered approach compared to previous studies aiming at improving REDs related symptoms in females (16). The knowledge and tools acquired by the athletes presumably enables a longer-lasting behavior change compared to studies where the participants are given nutritional supplements only (14). Other strengths of the present study are the use of validated screening tools, long-term follow-up, and inclusion of a control group, which have been lacking in previous studies (14, 15). In addition, hormonal contraceptive users were excluded, in order to get the true picture of menstrual function. This, however, complicates the recruitment of the participants since the prevalence of hormonal contraceptive users among endurance athletes have been reported to be as high as 68% (69). Although the participant information material described that hormonal contraception users could not participate, we had to exclude 28% of the athletes who had signed up for the study due to the use of hormonal contraceptives. Unfortunately, the exclusion of hormonal contraceptive users may have prevented potential REDs cases to participate, e.g., since hormonal contraceptives may mask underlying menstrual dysfunctions.

A limitation of the present study is the lack of long-term follow-up in the CON athletes. Consequently, the long-term effects of the FUEL intervention can only be speculative. By having long-term follow-up of the CON condition, athletes would have to wait an additional year before being offered the FUEL intervention, thereby increasing the risk of a higher drop-out rate in this group. There may also be ethical considerations, since these athletes all have REDs related symptoms, early intervention is important. While prioritizing the intervention in athletes' off-season, it is a limitation that data assessment was conducted at different phases of the athletic season for the intervention group and the control group, which may reduce comparability between the two groups.

The low number of participants in the CON group is also a limitation. Based on an initial analysis during the recruitment phase with an expected improvement in LEAF-Q score of 3 and type I and Type II error of 5% and 20% respectively, the power calculation suggested 28 subjects in each group, suggesting an insufficient number of CON athletes. In addition, the expected improvement in LEAF-Q score of 3 is theoretically founded based on a project group discussion, without any previous studies to lean on.

Although it would have been interesting to collect data via physical laboratory tests, e.g., including body composition and female sex hormones to verify menstrual status, this study and its measurement methods more closely reflect what is practically possible for most athlete-based centers where time and resources are a critical constraint. The intervention and methods used may therefore more easily be implemented in real life settings.

Despite that this study included a combination of online lectures and individual consultations using behavior change theories and approaches, intervening athletes alone may be insufficient for behavior change and thus changes in REDs related symptoms. As previously addressed (36), cultural revolutions and changes in social norms are needed, which involves inclusion of coaches, health professionals, entire teams/clubs, and relatives of the athletes. Hence, future research should aim for also including the athletes' entourage.

## 5. Conclusion

In this group of endurance athletes, participating in the FUEL intervention implies long-term improvement of REDs related symptoms, including menstrual function. In addition, short and long-term follow-up suggest no adverse effects on eating disorder symptoms. The lack of long-term follow-up for the CON condition indicates, however, that the results should be interpreted with caution. Nevertheless, the FUEL intervention seems promising as a part of management of REDs related symptoms in female endurance athletes.

## Data Availability

The raw data supporting the conclusions of this article will be made available by the authors, without undue reservation.
